# A model for genuineness detection in genetically and phenotypically similar maize variety seeds based on hyperspectral imaging and machine learning

**DOI:** 10.1186/s13007-022-00918-7

**Published:** 2022-06-11

**Authors:** Keling Tu, Shaozhe Wen, Ying Cheng, Yanan Xu, Tong Pan, Haonan Hou, Riliang Gu, Jianhua Wang, Fengge Wang, Qun Sun

**Affiliations:** 1 Department of Plant Genetics & Breeding and Seed Science, College of Agronomy and Biotechnology, Ministry of Agriculture and Rural Affairs/Beijing Key Laboratory of Crop Genetic Improvement, China Agricultural University/The Innovation Center (Beijing) of Crop Seeds Whole-Process Technology Research, Beijing, 100193 People’s Republic of China; 2 Beijing Key Laboratory of Vegetable Germplasm Improvement, Beijing Vegetable Research Center, Beijing Academy of Agriculture and Forestry Sciences (BAAFS), Beijing, 100097 People’s Republic of China; 3 Beijing Key Laboratory of Maize DNA Fingerprinting and Molecular Breeding, Maize Research Center, Beijing Academy of Agriculture and Forestry Sciences (BAAFS), Beijing, 100097 People’s Republic of China

**Keywords:** Maize seed, High-throughput, Phenotyping, Non-destructive testing, Varietal purity

## Abstract

**Background:**

Variety genuineness and purity are essential indices of maize seed quality that affect yield. However, detection methods for variety genuineness are time-consuming, expensive, require extensive training, or destroy the seeds in the process. Here, we present an accurate, high-throughput, cost-effective, and non-destructive method for screening variety genuineness that uses seed phenotype data with machine learning to distinguish between genetically and phenotypically similar seed varieties. Specifically, we obtained image data of seed morphology and hyperspectral reflectance for Jingke 968 and nine other closely-related varieties (non-Jingke 968). We then compared the robustness of three common machine learning algorithms in distinguishing these varieties based on the phenotypic imaging data.

**Results:**

Our results showed that hyperspectral imaging (HSI) combined with a multilayer perceptron (MLP) or support vector machine (SVM) model could distinguish Jingke 968 from varieties that differed by as few as two loci, with a 99% or higher accuracy, while machine vision imaging provided  ~ 90% accuracy. Through model validation and updating with varieties not included in the training data, we developed a genuineness detection model for Jingke 968 that effectively discriminated between genetically similar and distant varieties.

**Conclusions:**

This strategy has potential for wide adoption in large-scale variety genuineness detection operations for internal quality control or governmental regulatory agencies, or for accelerating the breeding of new varieties. Besides, it could easily be extended to other target varieties and other crops.

**Supplementary Information:**

The online version contains supplementary material available at 10.1186/s13007-022-00918-7.

## Background

Maize (*Zea mays* L.) is one of the most widely consumed crops worldwide, and represents a major source of food, livestock feed, and industrial raw materials [1, 2]. However, the recent, remarkable expansion of maize varieties has accompanied varietal infringement with inferior seeds or imitation varieties [3, 4]. In addition, lax control in seed production and processing has led to adulteration of commercial varieties and a decline in seed purity, for which had been reported that every 1% reduction in seed purity would reduce maize yield by 3.7–5% [5, 6]. Detection of variety genuineness and purity is therefore critically important to farmers and seed producers alike. Routine methods such as screening seedling morphology, isoenzyme electrophoresis, or simple sequence repeat (SSR) detection have advantages of high accuracy and reliability. Still, they also have disadvantages, such as being time-consuming, requiring highly specialized training, or being destructive to seeds [3, 7–9]. Exploring new appropriate strategies is urgently needed to meet current demands for accurate, high-throughput, cost-effective, and non-destructive detection of maize variety genuineness.

Machine vision is the most common and quickly adopted method for non-destructive testing of seed quality. It can classify seeds with different qualities by combining machine vision (typically RGB images) with machine learning algorithms that analyze the differences between seeds' phenotypic features (i.e., shape, color, and texture) [10–16]. However, this method is limited in distinguishing seeds from genetically and phenotypically similar lines. Fortunately, high-throughput phenotyping—hyperspectral imaging (HSI) may overcome this issue, which incorporates much spectral and spatial information simultaneously. This method can effectively differentiate and classify target objects or predict crop traits by detecting subtle differences in chemical composition and distribution [17–20]. Moreover, considerable evidence indicates that spectral characteristics are genotype-specific and can be used to distinguish plant genotypes [21], suggesting the feasibility of identifying crop varieties by hyperspectral imaging [17, 22–26].

Both machine vision and HSI obtain large phenotypic datasets, which require efficient data processing and statistical analysis, leading to machine learning algorithms to handle image analysis. The most common machine learning algorithms, random forest (RF) and support vector machine (SVM), have been successfully applied to a range of classification tasks [27–32]. In addition, multi-layer perceptron (MLP) has been broadly used for modeling and prediction in agricultural programs due to their high computational efficiency and accuracy [32–35].

**Fig. 1:**
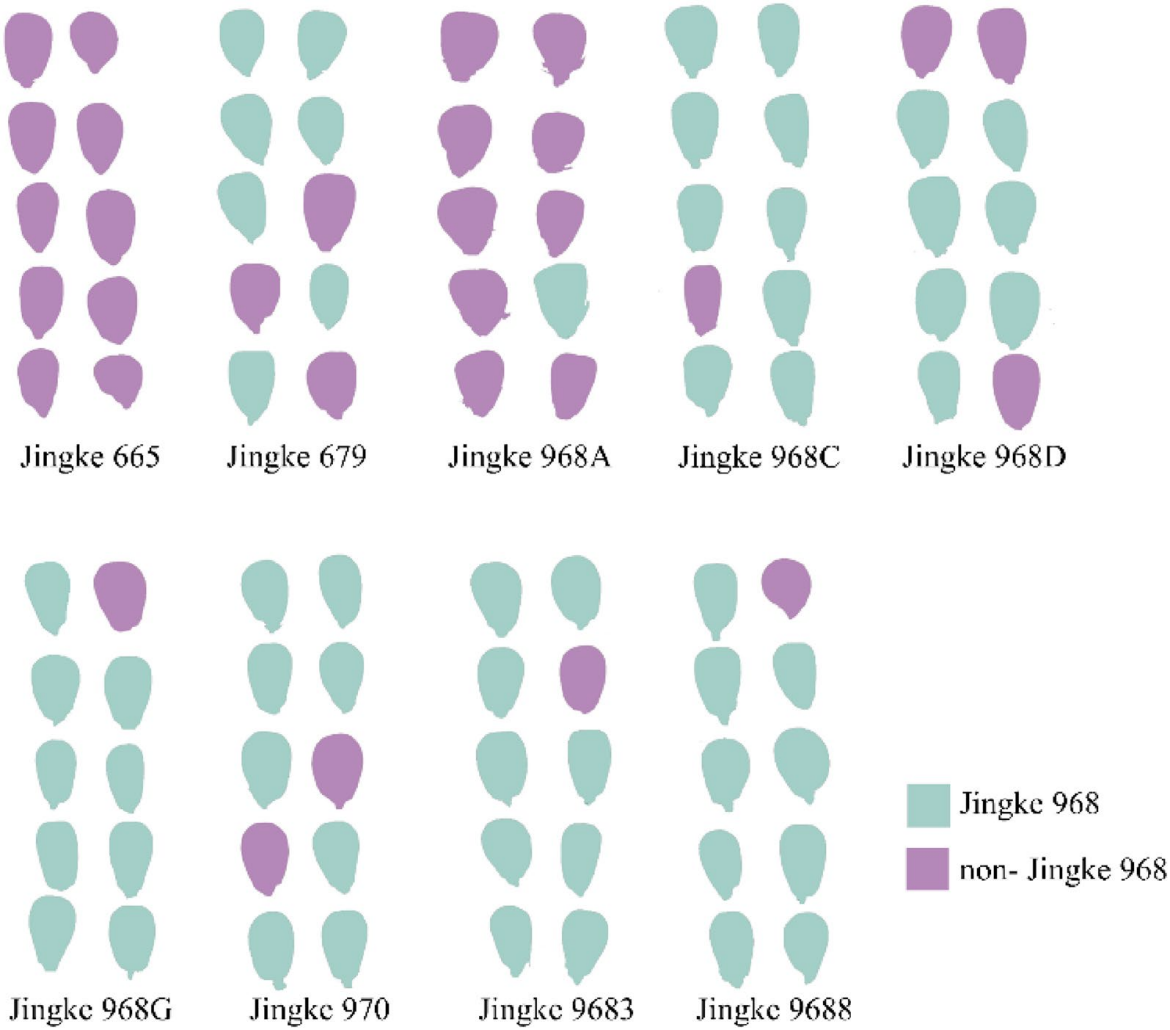
Variety genuineness detection result visualization. These nine genetically and phenotypically similar maize varieties (non-Jingke 968) of target variety Jingke 968 were tested using the model of Tu et al. [3] based on RGB images and the VGG16 network. Purple represents the detection result as non-Jingke 968. Blue means the detection result is Jingke 968

Previous studies reported successful non-destructive genuineness detection for target maize variety against regular commercial corn hybrids using machine vision with deep learning algorithms [3]. However, our further research found that this method was powerless against those genetically and phenotypically similar varieties. By combining RGB images and the VGG16 network, the established model was used to detect nine other genetically similar maize varieties of Jingke 968. The result indicated that except for variety Jingke 665 and Jingke 968A with higher recognition accuracy, most of the remaining seven varieties were incorrectly identified as Jingke 968. Then the overall recognition accuracy was as low as 34.4% (Fig. 1).

Hyperspectral image processing combined with machine learning algorithms has been used to classify the varieties of maize seeds according to differences in their chemical composition [26, 36–38]. More details for those varieties of classification tasks are shown in Table 1. Despite the success of this approach, these methods could only classify a limited variety of maize seeds, whereas discriminating between a large number of varieties not used in the training set presented a challenge to the performance of these applications. Moreover, none of these studies applied HSI to detecting maize variety genuineness. Consequently, we would turn this multivariate discriminant analysis-based classification method into a binary classification problem, that is, the detection of target variety and non-target varieties. The established model will still be effective for varieties other than the training set, and can identify and classify them as non-target varieties. Therefore, the variety genuineness detection model can still be carried out to maintain the high purity of the target variety.

**Table 1:** Applications of hyperspectral imaging in maize seed variety classification tasks

Number of cultivars	Varieties	Application	Result (%)	References
4 normal maize hybrids	Hangyunuo No.1, Suyunuo 14, Huyunuo No.1, and Yanhejin 2000	Variety identification	98.2	Yang et al. [36]
17 normal maize hybrids	BNA 07, SNN 12, Bositian 8, Gumang 178, Huayu 11, Jizaojinxiangnuo, Jinnuowang, Jinsaitian, Jingketian 195, Lianyu 16, Nongda 108, Sida 205, Wannuo 11, Xiangtiannianyumi, Yudan 998, Zhendan 958, and Zhongnongdatian 413	Variety identification	99.13	Xia et al. [37]
3 normal maize hybrids	Nonghua 213, Yinyu 274, and Yinyu 439	Variety identification	76.25	Shao et al. [38]
4 common maize varieties and 4 silage maize varieties	Datian 387, Quchen 8, Quchen 11, and Quchen 13; Quchen9, Quchen19, Quchen29, and Quchen513	Variety identification	~ 98	Bai et al. [26]

Here, we focused on Jingke 968, a predominant maize variety cultivated in China with high yield, desirable seed traits, multiple disease resistance, and wide environmental adaptability. Due to the high demand for Jingke 968 seeds, screening for genuineness and purity represents a problematic and potentially labor-intensive task that requires accuracy in discriminating similar phenotypes, efficiency in handling high seed volume, and cost-effectiveness relative to methods using expensive consumables [39].

To this end, we explored models that could work in common hybrids while also efficiently eliminating varieties genetically similar to that of the target variety. This strategy could also facilitate breeding programs for new varieties and resolve problems of variety adulteration. We obtained RGB and hyperspectral images from Jingke 968 (abbreviation: JK 968) and genetically similar non-Jingke 968 (abbreviation: non-JK 968) varieties and tested three machine learning algorithms for their ability to distinguish between varieties using only information extracted from these two image types. The specific objectives were as follows: (1) to establish a high-performance genuineness detection model for distinguishing genetically similar maize varieties based on seed phenotype with machine learning; (2) to compare machine vision and hyperspectral imaging for seed phenotype data collection to determine which imaging method is most appropriate for genuineness detection; (3) to compare the accuracy of different variety genuineness detection models for varieties not included in training data; (4) to establish a method for updating models to improve their ability to distinguish varieties not included in training data.

## Results

### Less efficient models for detection of variety genuineness based on machine vision

**Fig. 2:**
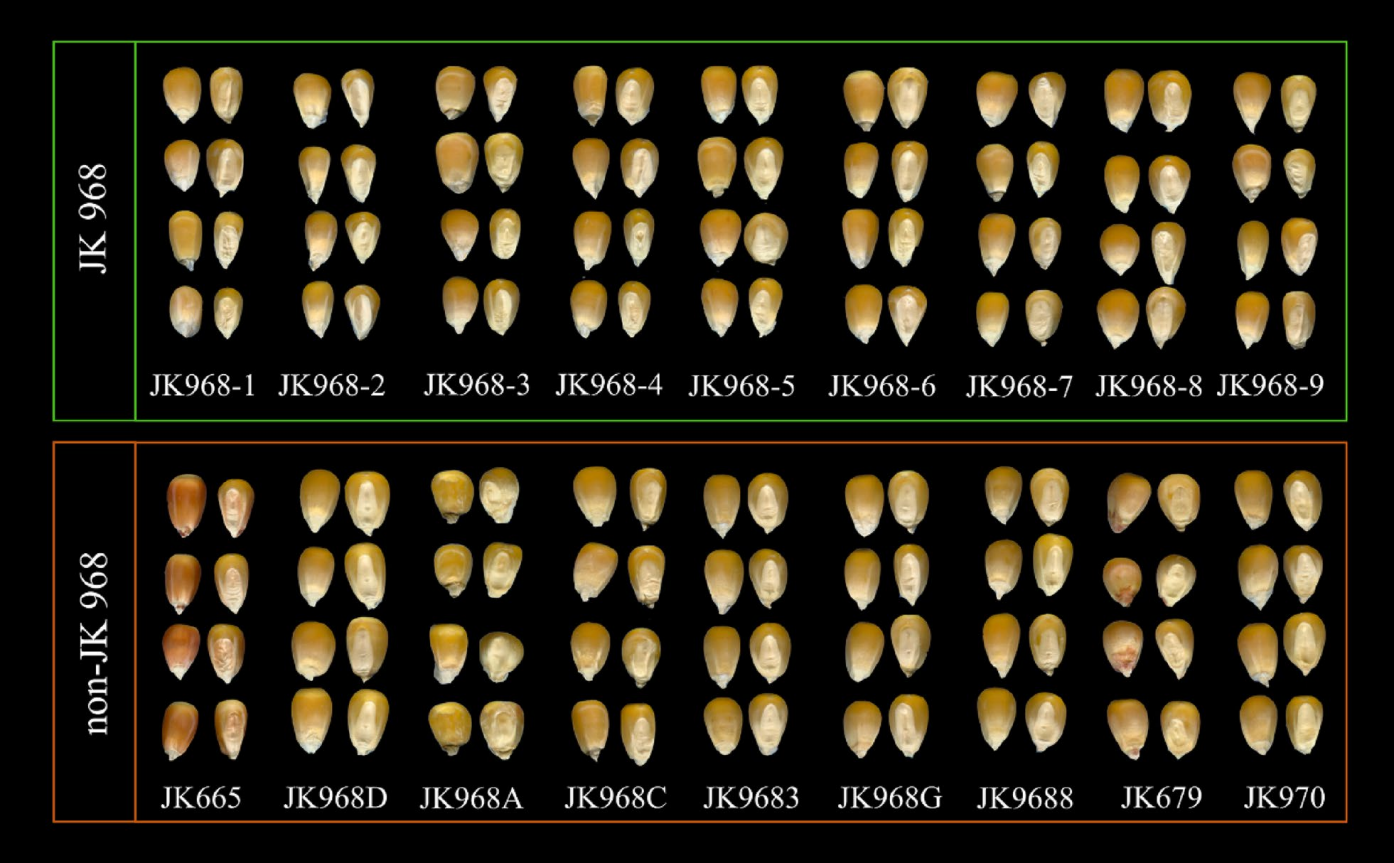
The germ and non-germ surfaces of maize seeds for different varieties. The top row represents nine seed lots of maize variety Jingke 968 for the JK 968 category. The bottom row represents the other nine non-target varieties for the non-JK 968 category, which are genetically similar to Jingke 968 variety

**Fig. 3:**
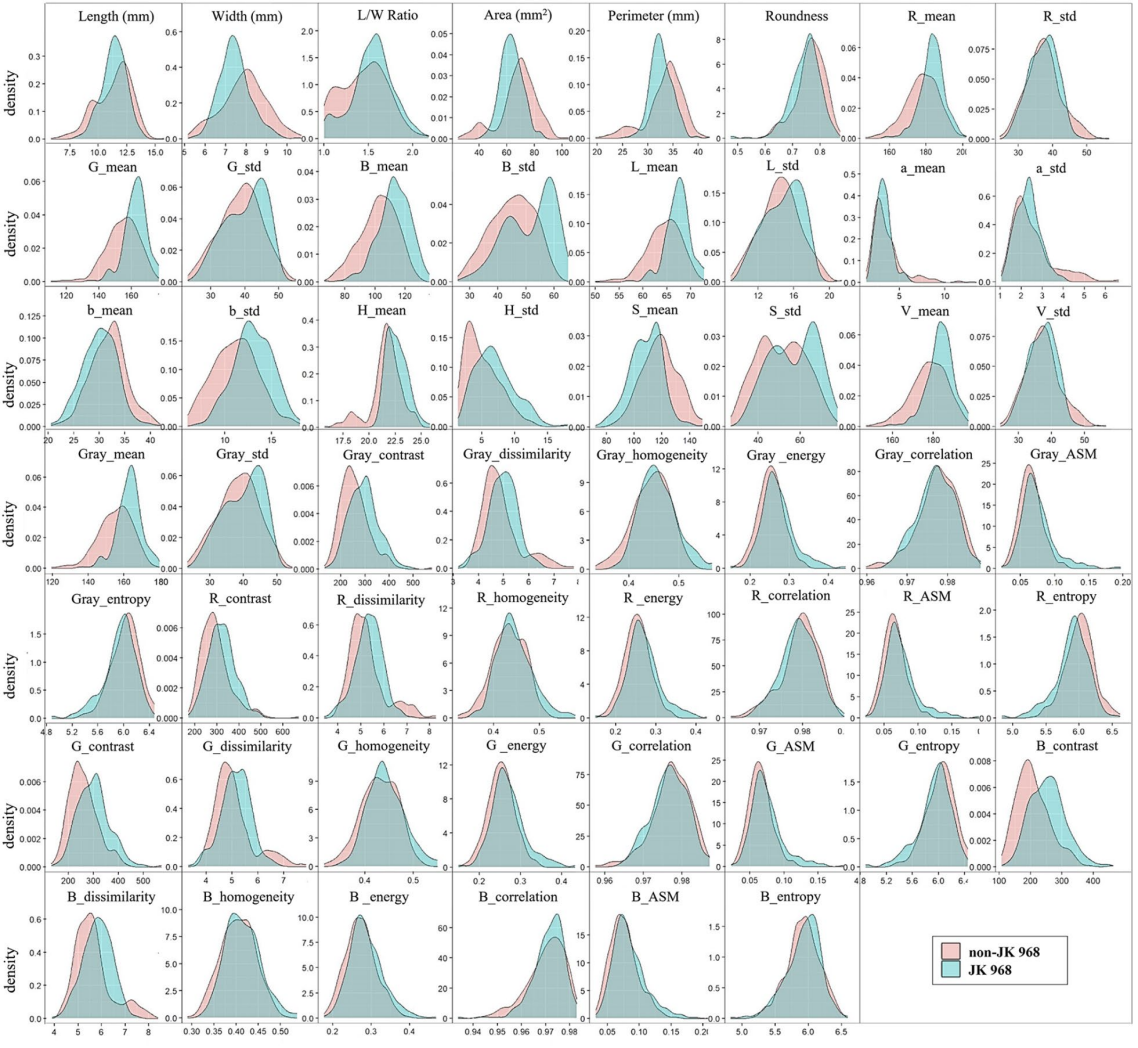
The probability density distributions of 54 features for JK 968 and non-JK 968, extracted from the seed germ surface

In order to develop a reliable high-throughput method for sorting the target seed variety from maize seeds of other genetically and phenotypically similar varieties, we first tested phenotypic RGB images with different modeling algorithms to evaluate their ability to distinguish image data of these two categories of maize kernels. As shown in Fig. 2, there is variability in seed appearance within and among JK968 seed lots, such as different sized JK 968-9 and smaller kernels in JK 968-2. Conversely, several genetically similar non-JK 968 varieties have a remarkably similar appearance to the target JK 968 variety (e.g., JK 968D, JK 968C, JK 9683, JK 968G, JK 9688, and JK 970). These similar varieties are thus indistinguishable purely through visual inspection. To identify differences between varieties, we then extracted 54 features, including shape, color, and texture features, from the germ and non-germ surfaces in the RGB image data of 315 JK 968 and 315 non-JK 968 maize seeds. Figure 3 plots the probability density distributions of these features from the germ surfaces of seeds. These features primarily overlapped between the two categories, indicating that some of these features were not informative for distinguishing JK 968 from non-JK 968. More sophisticated analytical methods may be necessary to sort them accurately. Furthermore, this confounding feature overlap was evident too in the probability density distribution plots of image data from non-germ seed surfaces (Additional file 1: Figure S1).

**Fig. 4:**
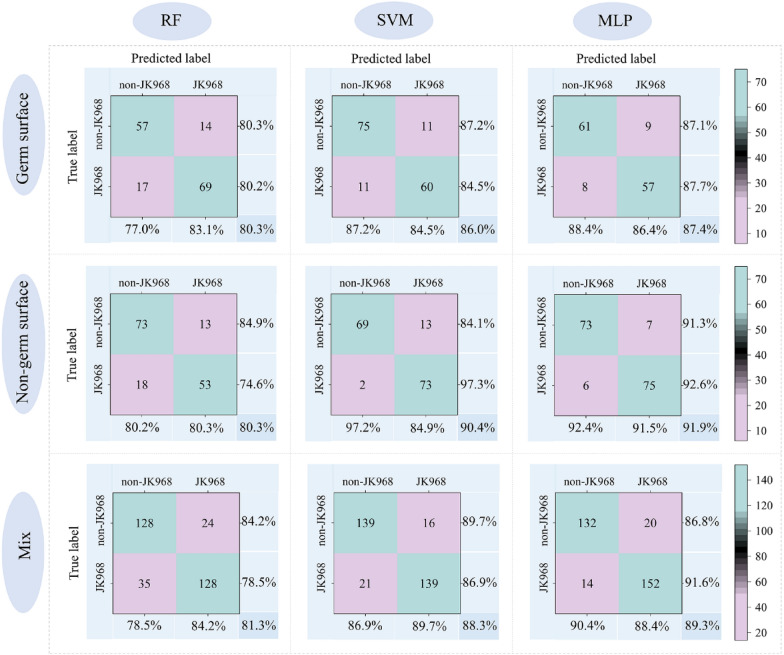
Confusion matrix of model detection results in the test set using machine vision information. The RF, SVM, and MLP models are presented in columns from left to right, respectively. Each row from top to bottom represents models developed using germ surface features, non-germ surface features, and a mixture of germ and non-germ features. The percentages in the lower right corners indicate the accuracy of each test set

Using these features, we assembled three datasets that included imaging data from germ surfaces, non-germ surfaces, and a mixture of the two. Then these three datasets were used as inputs for the RF, SVM, and MLP network models and established genuineness detection models for JK 968 against genetically similar varieties (Fig. 4). No significant differences were found in their detection performance, regardless of whether the data used in the input variables were obtained from the germ surface, the non-germ surface, or a mixture. However, we noted that the accuracy of the SVM and MLP models were both better than that of RF, although the overall accuracy remained low (i.e.,  ~ 90% accuracy for better models). These results thus indicated that machine vision image data alone was insufficient to establish an accurate and reliable model for genuineness detection for maize seeds, especially among highly genetically and phenotypically similar varieties.

### High-accuracy detection of variety genuineness by modeling HSI data

**Fig. 5:**
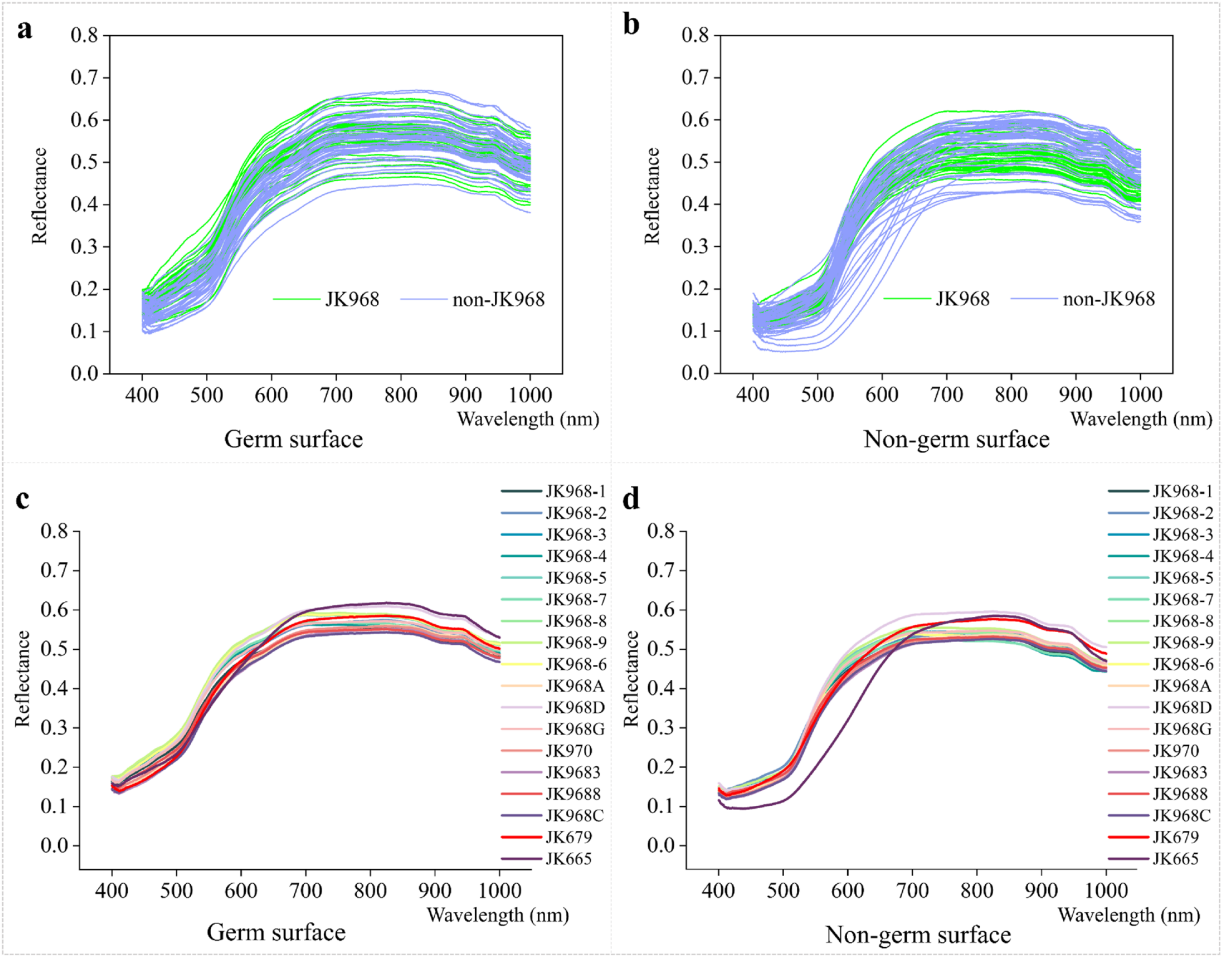
Raw spectra of JK 968 and non-JK 968 maize seeds obtained by the hyperspectral imaging system. **a**, **b** Spectra of individual maize seeds collected from germ or non-germ surfaces (Five grains were randomly selected from each seed lot or variety to show the distribution clearly). **c**, **d** Average spectra of every JK 968 seed lot and non-JK 968 variety obtained from the germ or non-germ surfaces

In order to improve the accuracy of genuineness detection for Jingke 968 seeds, we then explored whether VIS/NIR hyperspectral imaging could detect the subtle differences in spectral reflectance related to differences in chemical composition between varieties. After filtering out the noise signal, 756 variables between 400 and 1000 nm were retained as full wavelengths for use in subsequent analyses. The raw spectra of each maize seed's germ and non-germ surfaces are shown in Fig. 5a, b. The spectral reflectance for all maize seeds was less than 0.8, and the different varieties showed similar levels of variability within lots (Fig. 5c, d). However, some spectral curves differed between JK 968 and non-JK 968 seeds (about 700–1000 nm), while the spectral curves for the remaining wavelengths showed substantial overlap between varieties (especially 400–600 nm). This result thus showed that distinguishing between these genetically similar varieties with spectral data still presented difficulties for accuracy and efficiency.

**Fig. 6:**
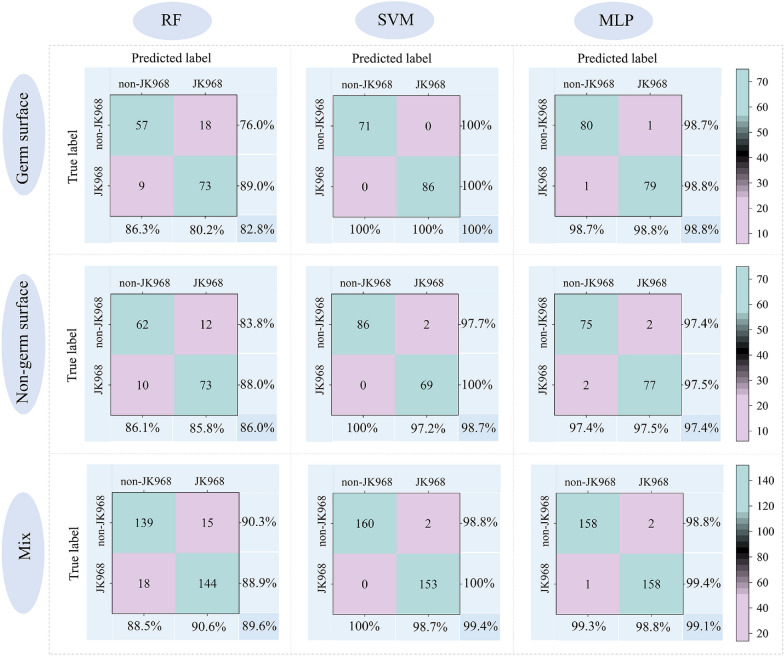
Confusion matrix of detection accuracy for models using hyperspectral reflectance data. RF, SVM, and MLP models are presented in columns from left to right. Each row from top to bottom represents models developed using reflectance of the germ surface, non-germ surface, or a mixture of germ and non-germ surfaces. The percentages in the lower right corners indicate the accuracy

We subsequently used the RF, SVM, and MLP algorithms to establish discriminant analysis models based on the 756 spectral bands of the germ surfaces, non-germ surfaces, and a mixture of the two. The results of test sets for each model showed apparent differences in accuracy between models, with the SVM and MLP models (both with the accuracy of mixture data-based model over 99%) performing significantly better than the RF model (accuracy lower than 90%) (Fig. 6). Notably, the MLP and SVM models showed comparably high accuracy in distinguishing varieties, with overall accuracy reaching approximately 100% in test sets. Similarly, we identified no significant differences in detection accuracy among the germ surface, the non-germ surface, or mixed dataset inputs.

**Fig. 7:**
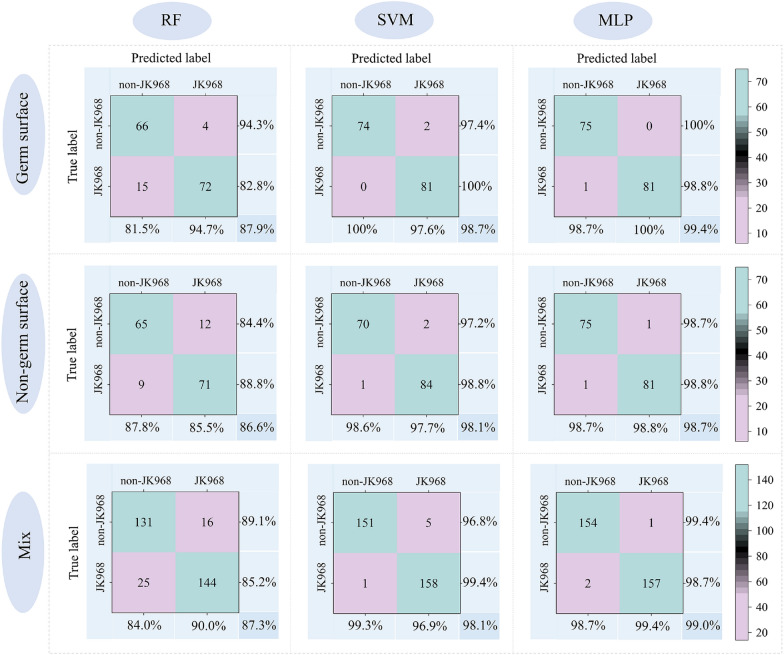
Confusion matrix of model detection results using characteristic features identified by SPA preprocessing. RF, SVM, and MLP machine learning models are respectively presented in columns from left to right. Each row from top to bottom represents models developed using reflectance values of the germ surface, non-germ surface, or a mixture of surface data. The percentages in the lower right corners indicate the detection accuracy in the test set

To reduce the computational burden, the wavelength selection algorithm, SPA, was applied to select the most informative spectral features (i.e., wavelengths) (Table 2). For the germ surface, the non-germ surface, and the mixed data set, 9, 11, and 10 wavelengths were selected. Then, these characteristic wavelengths were used to build detection models with the RF, SVM, and MLP algorithms (Fig. 7). Comparisons between algorithms revealed that SVM and MLP-based models showed consistently high accuracy, stabilizing at  ~ 99%, with no significant differences in performance between germ surface, the non-germ surface, and mixed data inputs. Based on these results, both SVM and MLP with mixed seed surface data were selected as the best models for detecting genuineness, regardless of whether input data included full spectra or only characteristic wavelengths.

**Table 2:** Characteristic wavelength selected using SPA

Spectral data sources	Effective wavelengths (nm)
Germ surface	405.5, 407.8, 409.2, 410.7, 414.4, 421.1, 454.7, 693.3, 919.0
Non-germ surface	404.8, 407.0, 407. 8, 409.2, 410.7, 412.2, 421.1, 488.7, 607. 5, 691.8, 919.0
Mixed surface data	401.8, 405.5, 407.8, 409.2, 410.7, 420.3, 492.5, 576.3, 817.8, 935.0

### Verification and update of the selected genuineness detection model based on HSI

**Fig. 8:**
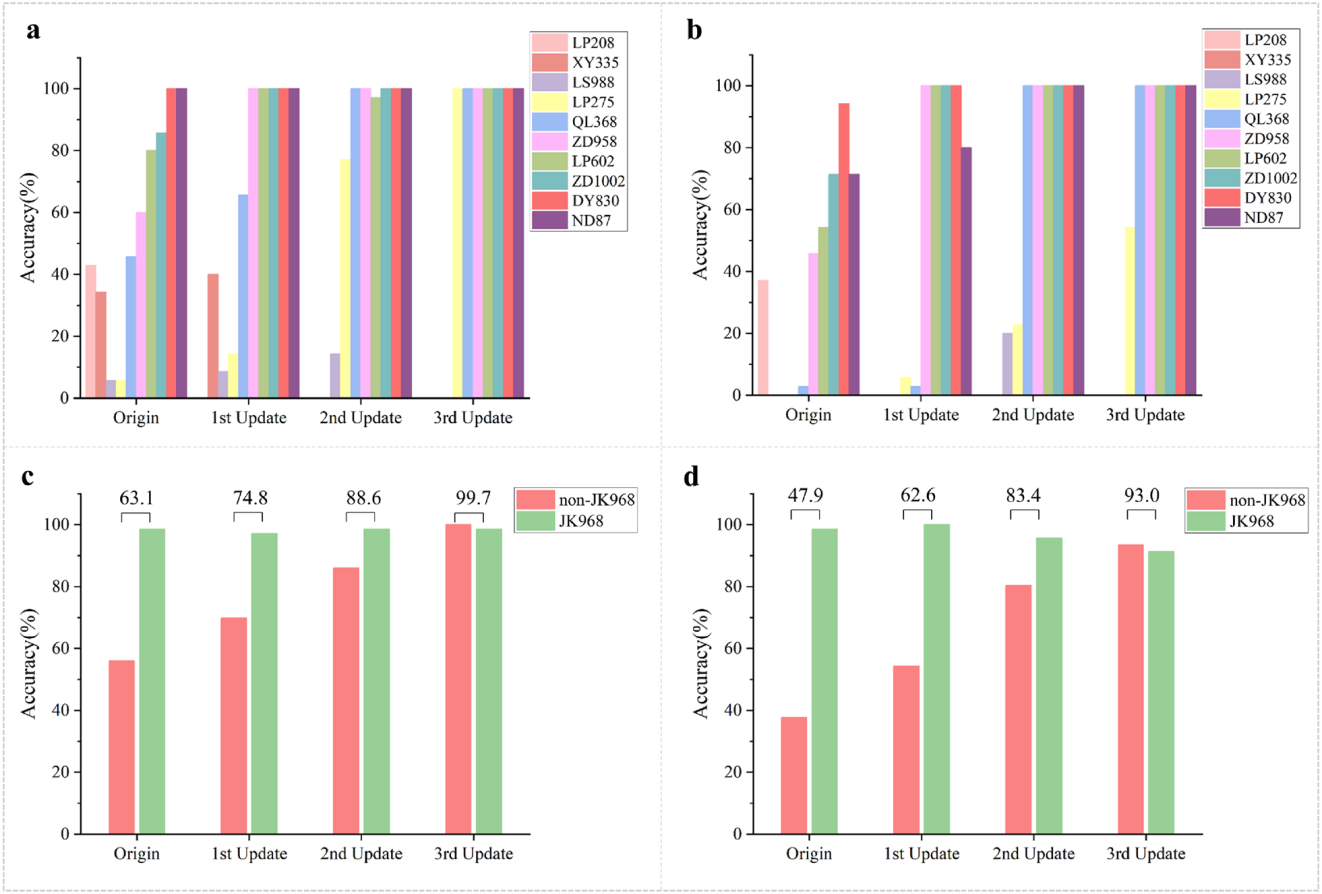
Increased recognition accuracy for JK 968 and several non-JK968 varieties through model updating. The MLP-based HSI mixed surface data genuineness detection model. **a** Histogram showing the recognition accuracy of all non-JK 968 varieties after each model update with full-spectrum hyperspectral reflectance data. **b** Histogram showing the recognition accuracy of all non-JK 968 varieties after each model update with characteristic wavelengths selected by SPA preprocessing. **c** Histogram of recognition accuracy for JK 968 and non-JK 968 seeds after model update with full hyperspectral reflectance data. **d** Histogram of the recognition accuracy of JK 968 and non-JK 968 seeds following each model update with characteristic wavelengths selected by SPA preprocessing. The numbers above each pair of columns represent the average detection accuracy for the model

In order to test the practicability and generalization of the SVM or MLP-based HSI mixed surface data models, we chose several common maize hybrids not used for model training to verify their performance. To this end, seventy JK 968 grains from two seed lots and 350 seeds from ten non-JK 968 common maize hybrids were selected for genuineness detection using either full spectra or ten effective wavelengths. As shown in Fig. 8, the results showed greater than 98% detection accuracy for JK 968 using either full spectra or ten features. However, among the ten non-JK 968 varieties not used for modeling training, the identification accuracy was higher for some (e.g., DY 830 and ND 87) but extremely low for others (e.g., LP 208, XY 335, LS 988, and others). Subsequently, we updated the MLP-mixed HSI model through an active learning strategy. The spectral information from varieties with recognition accuracy lower than 60% was added to the training data.

**Fig. 9:**
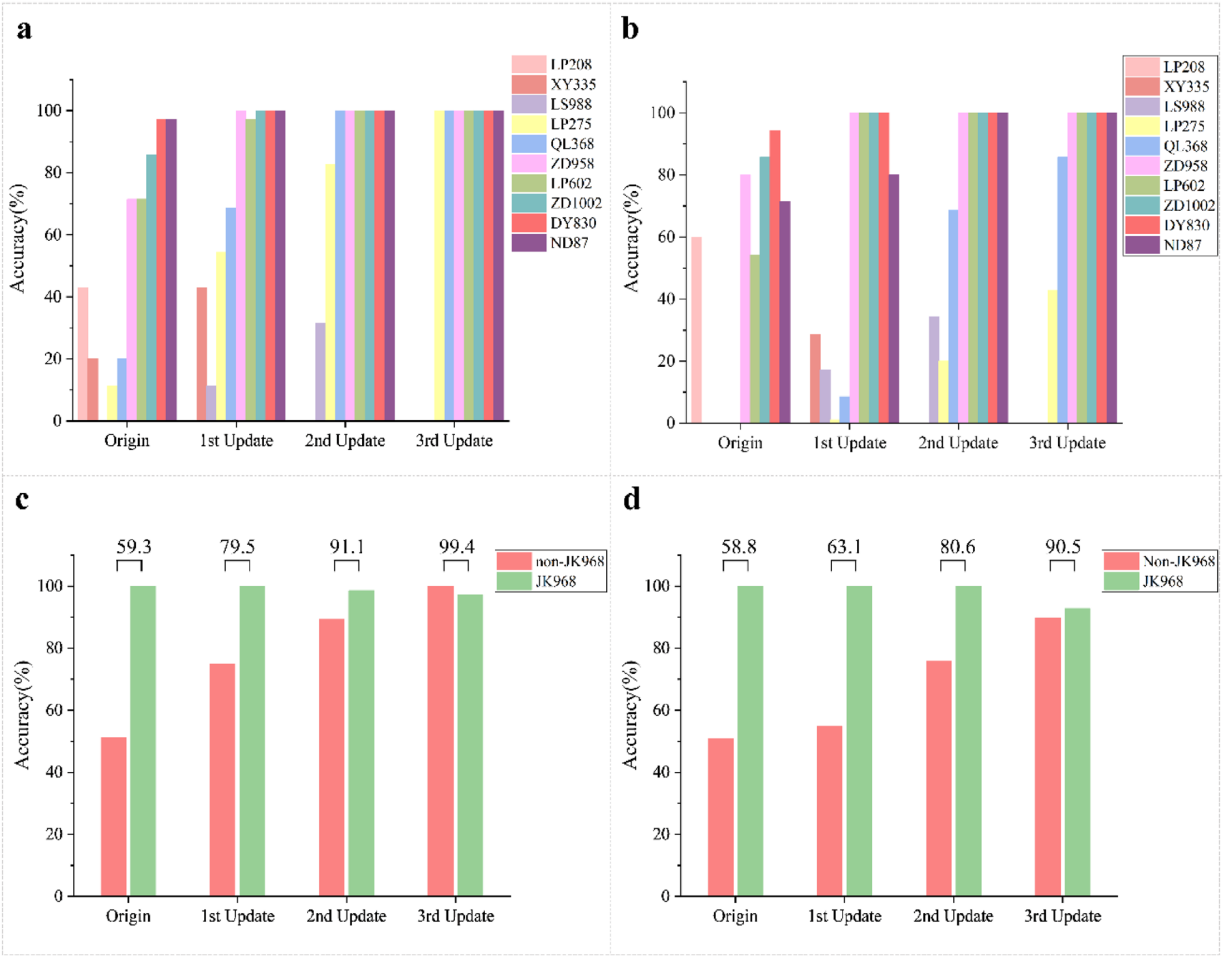
Increased recognition accuracy for varieties through model updating. The SVM-based HSI mixed surface data genuineness detection model. **a** Histogram showing the recognition accuracy of all non-JK 968 varieties after each model update with full-spectrum hyperspectral reflectance data. **b** Histogram showing the recognition accuracy of all non-JK 968 varieties after each model update with characteristic wavelengths selected by SPA preprocessing. **c** Histogram of recognition accuracy for JK 968 and non-JK 968 seeds after model update with full hyperspectral reflectance data. **d** Histogram of the recognition accuracy of JK 968 and non-JK 968 seeds following each model update with characteristic wavelengths selected by SPA preprocessing. The numbers above each pair of columns represent the average detection accuracy for the model

First, LP 208 was chosen for model updating. Then, the updated model was used to discriminate JK 968 seeds from those of the nine remaining non-JK 968 varieties. The results showed that the recognition accuracies for XY 335, LS 988, LP 275, ZD 958, QL 368, LP 602, and ZD 1002 were improved by 5.7, 2.9, 8.8, 40, 20, 20, and 14.3%, respectively, following the first update, while the DY 830 and ND 87 detection remained at 100% (Fig. 8). Next, XY 335 and LS 988 were randomly selected from the varieties with recognition accuracy that remained lower than 60% after the first update. Their mixed HSI surface data were added to the training data for the second and third model updates, respectively. Detection assays indicated that recognition accuracy again substantially improved for the other varieties, especially in the model based on full HSI spectra. Ultimately, the average recognition accuracy of the full spectrum model was improved to 99.7%, while the model's accuracy of using characteristic wavelengths increased to 93.0%, showing improvements of 36.6% and 45.1% over that of the original model, respectively. The model based on the SVM algorithm presented almost the same pattern (Fig. 9), for the average recognition accuracy of the full spectrum model was increased from 59.3 to 99.4%, and that using characteristic wavelengths raised from 58.8 to 90.5%, with improvements of 40.1% and 31.7% over that of the original model, respectively.

Taken together, these results indicated that the SVM or MLP-based genuineness detection model using full HSI spectra could exhibit the highest performance. They could distinguish between highly phenotypically similar seeds from genetically close cultivars. Still, they could also be extended (through relatively simple updates) to recognize common hybrids not included in training data with over 99% accuracy.

## Discussion

In recent years, infringement and adulteration of maize varieties have been a frequent occurrence, influencing the grain yield. Therefore, it is essential to detect the variety genuineness and purity. Given some shortcomings of traditional detection methods, we intended to explore an appropriate modeling strategy based on seed phenotype and machine learning, to meet the urgently current needs for accurate, high-throughput, cost-effective, and non-destructive detection of maize variety genuineness.

It is well known that seeds of the same variety may exhibit phenotypic differences due to storage conditions, cultivation year, and environmental conditions, which can confound the recognition of target varieties and further affect the model’s accuracy [2]. Therefore, we collected as many seed lots of target varieties as possible to ensure the generalizability of the models tested here. Moreover, it is challenging to detect only one specific side of seeds. To address these issues, we analyzed separate and mixed data from the germ and non-germ surfaces and found no significant difference in recognition accuracy. Hence, phenotypic data can be obtained randomly from any seed surface, improving the operation time and efficiency, consistent with the conclusion of Tu et al. [3]. When tested with genetically similar varieties, machine vision combined with machine learning algorithms showed apparently low accuracy, and failed to meet the variety genuineness and purity testing requirements. We also tested the method of Tu et al. [3], which directly used the seed images as input for the VGG16 network to distinguish between JK 968 and non-JK 968 seeds. Unfortunately, the detection accuracy was as low as 60% due to the highly similar seed morphology in RGB images between the target JK 968 and several genetically similar varieties.

Hyperspectral imaging, which can reflect subtle differences in the chemical composition of seeds from genetically similar varieties, was then used to establish a highly accurate detection model for variety genuineness. Differences in spectral information among different varieties at the same wavelength were similar in this study to those in previous reports [18, 37]. In particular, the reflectance values were not the same between different varieties, although the spectral curves of these different varieties showed the same trend [40]. For maize seeds, the absorbance of the spectra at 400–500 nm is proportional to protein content, whereas the absorption peak between 500 and 750 nm is mainly attributable to the abundance of starches, oils, and other chemical compounds [41]. The peak near 980 nm was shown to be the central absorption wavelength for the second overtone of O–H stretching, caused by the presence of water and carbohydrates [42]. The presence of similarities or differences in starch content, carbohydrates, and other components thus form a reliable basis for using hyperspectral imaging to distinguish different maize varieties [2, 37].

However, it should be noted that HSI spectra frequently overlap due to similarities in the composition of the seed epidermis. To resolve this issue, it was necessary to establish discriminant analysis models that fully exploit the available spectral variables to classify target seeds from those of other varieties. Besides, it is widely known that the similarities among varieties will affect classifiers’ performance [43]. In this study, the genuineness detection model based on SVM or MLP with full HSI wavelength data showed accuracy as high as 99% in sorting complex samples, including several JK 968 seed lots from different conditions and seeds of several other varieties genetically similar to JK 968. This accuracy reflects the advantages of hyperspectral data compared to that obtained by machine vision. The main advantage of RGB is a lower cost instrument system and faster image acquisition, compared with HSI. However, HSI offers hundreds or thousands of spectral bands rather than the reflectance in 3 spectral regions (Red, Green, and Blue), and thus contains more information on samples to improve discrimination performance [43, 44]. It should also be noted that real-time detection of maize seeds using full wavelength data remains challenging, owing to limitations in the speed of HSI data acquisition and processing [37]. Therefore, building a robust model based on a small number of characteristic features is necessary to reduce the related costs and prediction time [45]. After selecting a limited set of characteristic wavelengths using the SPA algorithm, HSI-based models still performed better than machine vision-based models by detecting informative peaks that indicate differences in starch, protein, water, and other components.

When tested with untrained samples, a well-established model with reliable performance is still expected to lose its effectiveness. To accommodate these samples, model updating is essential. We found that the addition of several untested varieties with low recognition accuracy to the training data greatly enhanced model performance in recognizing other untested varieties. Expanding the samples used to train the original model with seeds from the lowest accuracy samples can enable rapid updating of the model. It resulted in improved stability and adaptability (and consistently high performance) in recognizing a more comprehensive range of seed lots or varieties than in the original training data. We also found that with an increase in common hybrid varieties, the performance of the SVM or MLP model using full wavelength data performed significantly better than that using characteristic feature wavelengths. As shown in the study of Yang et al. [46], the prediction accuracy of sugarbeet seeds SVM model based on 16 characteristic wavelengths reduced by 3.18% than that of full wavelength. This difference in performance could be related to the selection of feature wavelengths from the original training data, which could overlook informative wavelengths needed to discern other varieties [24, 47]. Consequently, whether to use an algorithm such as SPA to select characteristic wavelengths should consider the actual application situation, which depends on the processing power of the computer, as well as the trade-off between the accuracy, rapidity, and generalization of the detection model. After all, the high-performance computer may significantly increase the related budgets.

Previous studies have researched the non-destructive identification of seed varieties based on hyperspectral imaging and machine learning or deep learning [4, 9, 17, 18, 24–26, 48, 49]. Although there might be a certain distance from the actual application due to a limited number of varieties in the training set, the successes of these studies guide the seed variety genuineness detection to ensure seed purity. These studies show that the SVM and deep learning models are effective algorithms for processing large phenotypic spectral datasets. Even though the convolutional neural network (CNN) performs slightly better than the SVM in some cases, their overall performances are very close [49]. So, we chose the traditional machine learning algorithms (SVM, MLP, and RF) and got detection accuracy above 99% (SVM and MLP) that can be widely adopted, especially in resource-limited agricultural settings. Usually, the discrimination result declined with the increase in the number of seed varieties. When seed varieties increased from two to four, the final discrimination accuracy of the SVM model dropped from 95.67 to 92.56% [48]. Consequently, we would turn this multivariate discriminant analysis-based classification method into a binary classification problem: the detection of target variety and non-target varieties. It can effectively deal with more varieties within or outside the training dataset, meanwhile maintaining high accuracy.

This novel strategy for rapidly detecting maize variety genuineness, which combines seed phenotype with machine learning algorithms, thus provided encouraging results for discriminating seeds from complex samples with highly similar varieties. RGB imaging coupled with deep learning enabled the detection of Jingke 968 genuineness in samples containing other normal corn hybrids, but phenotypically and genetically distant (that is, relatively easy) [3]. Furthermore, this current study shows the capacity for distinguishing Jingke 968 seeds in complex samples containing highly genetically similar varieties (i.e., varieties differing at only two loci) by integrating HSI data with SVM and MLP modeling. In addition, our research group is currently advancing this method by developing an intelligent and automatic variety genuineness detection system. It is currently in the testing stage and is expected to be used in the high-throughput online seed detection and selection system.

## Conclusion

In conclusion, limitations in the current methods for detecting variety genuineness have prevented automation and high-throughput identification of seed purity. This study represents, to our knowledge, the first description of a method for variety genuineness detection based on SVM or MLP modeling with hyperspectral imaging data. Our results indicate that this method is a rapid, highly accurate, and non-destructive tool for sorting seeds of a specific variety from those of other, highly genetically similar varieties. In particular, this model showed as high as 99% accuracy in discriminating between seeds from maize varieties differing at only 2–10 out of 40 SSR detection loci. In addition, genuineness detection using full wavelength data provided the highest accuracy of the models tested here, in samples containing genetically similar seeds or common hybrids not used in the training samples.

Moreover, the model shows extensive adaptability, and can be updated to accommodate varieties outside of the original training set through an active learning strategy. Based on its advantages of high accuracy and non-destructive imaging, this approach could have a wide range of applications in seed purity testing, seed genotyping, and intellectual property protection, as well as ensuring that the expected varieties are indeed deployed in the field. This approach can also be broadly applied to other crops for phenotypic correlation analyses to accelerate plant breeding programs via non-destructive testing.

## Methods

### Experimental samples

There were two maize seed groups, ‘JK 968’ and ‘non-JK 968’. For the JK 968 category, the target variety Jingke 968 contained nine seed lots from different years and producing areas, provided by several seed companies. The details of Jingke 968 seed lots were showed in Additional file 1: Table S1. Nine genetically and phenotypically similar non-target varieties, provided by Maize Research Center, Beijing Academy of Agriculture and Forestry Sciences, the breeding institution of maize variety 'Jingke 968', were considered non-JK 968 category. According to the SSR-based detection standard in China, they differ from Jingke 968 variety at as few as two to ten loci in 40 detection loci. Because these precious breeding materials were in limited quantities, and to solve the imbalance problem of samples, thirty-five maize seeds were selected from each Jingke 968 seed lot or non-Jingke 968 variety, so there were 315 seeds in each category. The details are shown in Table 3. Subsequently, Fig. 2 shows an overview of different maize varieties by scanned seed images.

**Table 3:** Numbers of seeds included in training data of nine different Jingke 968 seed lots and nine non-Jingke 968 varieties

Category	Variety	Year	Abbreviation	Number of different loci (Detected in 40 SSR loci)	Number of seeds
JK 968	Jingke 968	2019	JK968-1	/	35
	Jingke 968	2019	JK968-2	/	35
	Jingke 968	2020	JK968-3	/	35
	Jingke 968	2020	JK968-4	/	35
	Jingke 968	2020	JK968-5	/	35
	Jingke 968	2020	JK968-6	/	35
	Jingke 968	2020	JK968-7	/	35
	Jingke 968	2020	JK968-8	/	35
	Jingke 968	2020	JK968-9	/	35
Non-JK 968	Jingke 665	2016	JK665	2	35
	Jingke 968D	2020	JK968D	3	35
	Jingke 968A	2018	JK968A	5	35
	Jingke 968C	2020	JK968C	5	35
	Jingke 9683	2018	JK9683	7	35
	Jingke 968G	2020	JK968G	8	35
	Jingke 9688	2018	JK9688	8	35
	Jingke 679	2019	JK679	8	35
	Jingke 970	2018	JK970	10	35

Furthermore, seventy JK 968 grains and 350 seeds from ten common maize hybrids (non-JK 968) were randomly selected, from seed lots that did not participate in the model training, to verify the JK 968 genuineness detection model. The details for those seeds are shown in Table 4. All seeds were dried to about 10.0% of moisture content and stored at room temperature (25 ℃, RH 30%).

**Table 4:** External verification sample arrangement

Category	Variety	Year	Abbreviation	Number of seeds
JK 968	Jingke 968	2020	JK 968	35
	Jingke 968	2020	JK 968	35
Non-JK 968	Longping 208	2017	LP 208	35
	Longping 275	2017	LP 275	35
	Longping 602	2017	LP 602	35
	Zhengdan 958	2017	ZD 958	35
	Zhengdan 1002	2017	ZD 1002	35
	Qiule 368	2017	QL 368	35
	Xianyu 335	2017	XY 335	35
	Lianshu 988	2017	LS 988	35
	Dingyou 830	2017	DY 830	35
	Nongda 87	2017	ND 87	35

### Phenotypic evaluations

#### RGB image acquisition and feature extraction

For all seed samples imaged directly, no preconditioning is required. In the machine vision part, the scanner (Microtek scanmaker i360) with a CCD camera was used to obtain scanned images from maize seeds’ germ and non-germ surfaces. Tagged image file format (TIFF) images for the R, G, and B color channels were saved, measuring 2297 × 3381 pixels (h × w), and with a resolution of 300 dpi. To simplify the operation process and improve the efficiency of obtaining images, we scan hundreds of seeds simultaneously, but to ensure that the seed samples do not touch each other.

Six hundred thirty images of germ and non-germ surfaces were obtained from seeds in both the JK 968 and non-JK 968 categories. A total of 54 color, shape, and texture features for a single seed were extracted using Phenoseed (a software program developed by our lab and Nanjing AgriBrain Big Data Technology Co, Ltd.). The dataset of two categories was randomly divided into the training set and test set at the ratio of 3:1, to build a model with excellent generalization and robustness [50].

#### Hyperspectral reflectance data collection

In the hyperspectral imaging portion, we focused on the visible (VIS) and near-infrared (NIR) spectral reflectance bands. Each maize seed’s hyperspectral image was collected using a proto-type VIS/NIR hyperspectral imaging system with the wavelength range of 311–1090 nm, installed at the Beijing Key Laboratory of Crop Genetic Improvement, China Agricultural University. A detailed description of the whole system and parameters was available in the article of Zhang et al. [47]. All the hyperspectral image calibration and reflectance data extraction were then implemented by the HSI Analyzer software (Isuzu Optics Corp, Hsinchu, Taiwan, China).

Since the reflectance bands at both ends of the hyperspectral reflectance spectrum are significantly impacted by stochastic noise, 311–400 nm and 1000–1090 nm were removed from the original data. The process of spectral reflectance extraction is presented in Additional file 1: Figure S2. Every spectral curve represents the average reflectance of one maize seed. Consequently, 765 reflectance data between 400 and 1000 nm of each seed were considered input variables in further analysis. The ratio of the training set and test set was also set to 3:1.

There are high-dimensional data and much redundant information in the hyperspectral image. Dimensionality reduction and finding characteristic wavelengths are effective methods for hyperspectral data processing [18]. Therefore, applying the variable selection method to the analysis and processing of hyperspectral data is meaningful. One common way to select variables is the successive projections algorithm (SPA) approach, selecting several typical characteristic wavelengths that predict the output, without mathematical transformations on the raw reflectance data [18]. As a forward selection method, SPA is based on the principle of root mean square error (RMSE) minimization [46, 51]. It selects the variable with the lowest collinearity and redundancy. This study chose SPA to select a few sensitive wavelengths with smaller RMSE as characteristic wavelengths, through multiple linear regression analysis of full wavelength for maize seeds.

### Data-driven modeling

**Fig. 10:**
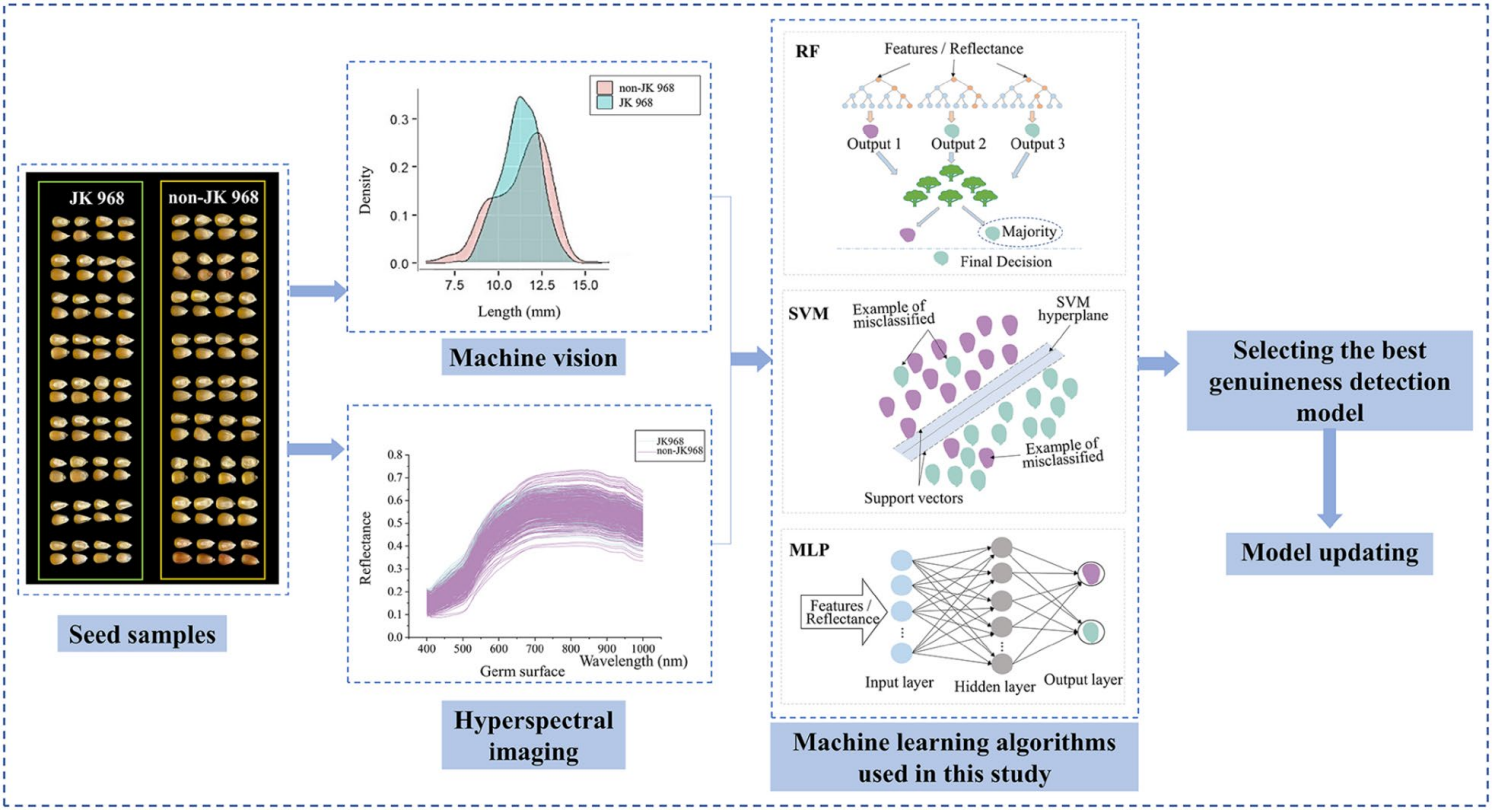
Technical route. *RF* random forest; *SVM* support vector machine; *MLP* multilayer perceptron

As shown in Fig. 10, random forest (RF), support vector machine (SVM), and multi-layer perceptron (MLP) were chosen and used for detecting seed genuineness of maize variety JK968, which were the most commonly used algorithms in previous studies [21, 52–56]. RF uses the decision tree as the base classifier to resample the same data set and establish multiple similar base classifiers. The classification prediction results of these base classifiers with slight differences can output the overall classification results by using integration methods such as averaging or voting [46]. This study used the RBF kernel to construct a nonlinear SVM model in the spectral analysis [17, 51]. It carried out the five-fold cross-validation operation and grid search program to calculate optimal penalty coefficient *c* and the kernel parameter *g*. The searching range was both set to − 10 to 10 with the step of 0.2. For the MLP network, we selected the sigmoid transfer function in the hidden layer and adopted the softmax activation function for the output layer, to achieve a binary classification task of variety genuineness.

### Model verify and update

It was proved that updating the training set was an effective method for model updating, improving the performance of developed models [2]. To increase the generalization of the genuineness detection model, we would update the model through an active learning strategy. The spectral information from varieties with recognition accuracy lower than 60% was added to the training data. Then, the updated model was used to detect the remaining external verification samples. The next variety with low recognition accuracy would be added to the training set for another update until the overall detection accuracy for external verification was improved to about 99%. Several varieties with reliable labeled as representative samples were extended to the original training set to increase the representativeness of the training set, which thus could significantly improve the model performance, reducing time and cost consumption.

### Analyzing

The SPA pretreatment, RF modeling, and SVM modeling were realized in Matlab (R2019a, The MathWorks, Inc.). The MLP modeling process was implemented efficiently in IBM SPSS Statistics 25. Training set and test set data were randomly split for every training with a ratio of 3:1. All the relevant parameters in each machine learning algorithm are optimized according to the input variables. The accuracy of the test set (the average of ten runs for each model) was selected as the evaluation indicator of the qualitative model. The OriginPro 2021 software and the ggplot 2 packages in the R 3.6.1 were used to visualize the results.

## Supplementary Information

**Additional file 1: Figure S1.** The probability density distributions of 54 features for JK 968 and non-JK 968, extracted from the non-germ surface. **Figure S2.** Spectral reflectance extraction. Step **a** Visual hyperspectral of the maize seeds from the HSI Analyzer software. Step **b** a binary mask, which only contains seeds with zero values for background, was acquired by threshold segmentation. Step **c**: the true regions of maize seeds from the image of 765 bands (400–100 nm) were segmented by the binary mask. Step **d** the mean spectral features of each maize seed were extracted in 765 bands to characterize the seeds. **Table S1.** The details of different Jingke 968 seed lots.

**Additional file 2: **The training data of hyperspectral imaging.

**Additional file 3: **The training data of machine vision.

**Additional file 4:** Machine learning code.

## Data Availability

All data (Additional file 2 and 3) generated or analyzed during this study and corresponding code (Additional file 4) are included in the Additional files.
