# Supplementary material for: A model for genuineness detection in genetically and phenotypically similar maize variety seeds based on hyperspectral imaging and machine learning
Source: Plant Methods. 2022 Jun 11;18:81. doi: 10.1186/s13007-022-00918-7 (PMC9188178; doi:10.1186/s13007-022-00918-7)
Supplement: Supplementary file 1 — Additional file 1: Figure S1. The probability density distributions of 54 features for JK 968 and non-JK 968, extracted from the non-germ surface. Figure S2. Spectral reflectance extraction. Step a Visual hyperspectral of the maize seeds from the HSI Analyzer software. Step b a binary mask, which only contains seeds with zero values for background, was acquired by threshold segmentation. Step c: the true regions of maize seeds from the image of 765 bands (400–100 nm) were segmented by the binary mask. Step d the mean spectral features of each maize seed were extracted in 765 bands to characterize the seeds. Table S1. The details of different Jingke 968 seed lots. [file 13007_2022_918_MOESM1_ESM.docx]

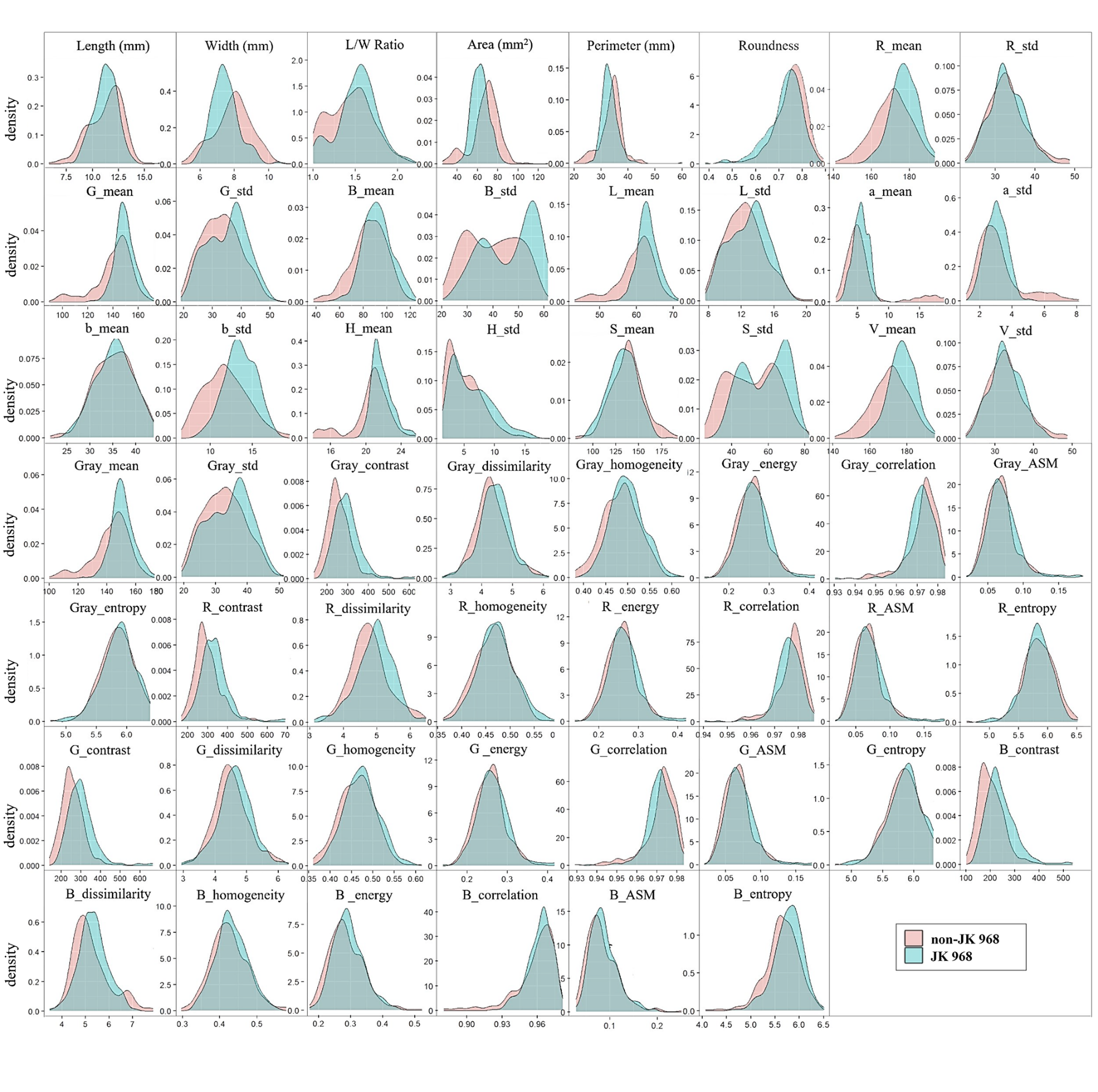


**Figure S1.** The probability density distributions of 54 features for JK 968 and non-JK 968, extracted from the non-germ surface.


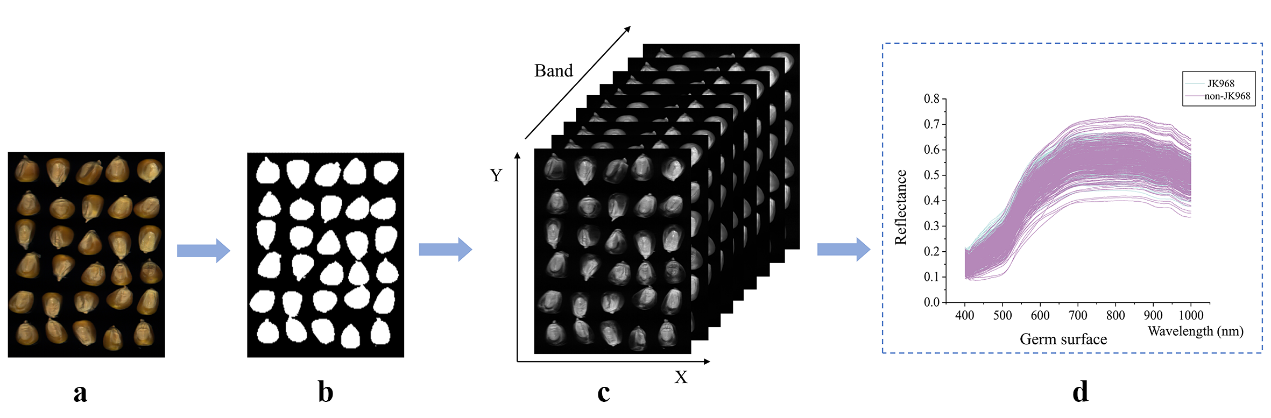


**Figure S2.** Spectral reflectance extraction. Step **a**: Visual hyperspectral of the maize seeds from the HSI Analyzer software. Step **b**: a binary mask, which only contains seeds with zero values for background, was acquired by threshold segmentation. Step **c**: the true regions of maize seeds from the image of 765 bands (400-100 nm) were segmented by the binary mask. Step **d**: the mean spectral features of each maize seed were extracted in 765 bands to characterize the seeds.

**Table S1 The details of different Jingke 968 seed lots**

| Category | Seed lot | Year | Abbreviation | Provider |
| --- | --- | --- | --- | --- |
| JK 968 | Jingke 968 | 2019 | JK968-1 | Shunxin Agriculture Sciences Co., Ltd |
|  | Jingke 968 | 2019 | JK968-2 | Shunxin Agriculture Sciences Co., Ltd |
|  | Jingke 968 | 2020 | JK968-3 | Shenzhou Lvpeng Agricultural Science & Technology Co., Ltd |
|  | Jingke 968 | 2020 | JK968-4 | Zhangye Jinyu Seed Industry Co., Ltd |
|  | Jingke 968 | 2020 | JK968-5 | Zhangye Jinyu Seed Industry Co., Ltd |
|  | Jingke 968 | 2020 | JK968-6 | Beijing Doneed Seed Co., Ltd |
|  | Jingke 968 | 2020 | JK968-7 | Zhangye Duocheng Agriculture Co., Ltd |
|  | Jingke 968 | 2020 | JK968-8 | China Forestry Group Zhangye Jinxiang Seed Co., Ltd |
|  | Jingke 968 | 2020 | JK968-9 | Zhangye Golden Sunflower Seed Industry Co., Ltd. |
